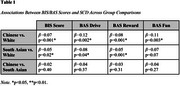# Ethnicity Moderates the Associations between Personality‐Related Motivational Systems and Subjective Cognitive Decline

**DOI:** 10.1002/alz70861_108822

**Published:** 2025-12-23

**Authors:** Angelina Zhang, Simran Malhotra, Rohina Kumar, Sarah‐Mei Chen, Rachel Yep, Katie L Vandeloo, Tulip Marawi, Harleen Rai, Alexander J Nyman, Georgia Gopinath, Madeline Wood Alexander, Silina Z Boshmaf, Walter Swardfager, Sandra E. Black, Maged Goubran, Jennifer S Rabin

**Affiliations:** ^1^ Hurvitz Brain Sciences Program, Sunnybrook Research Institute, Toronto, ON Canada; ^2^ Rehabilitation Sciences Institute, University of Toronto, Toronto, ON Canada; ^3^ Department of Pharmacology and Toxicology, University of Toronto, Toronto, ON Canada; ^4^ Division of Neurology, Department of Medicine, Sunnybrook Health Sciences Centre, Toronto, ON Canada; ^5^ Department of Medical Biophysics, University of Toronto, Toronto, ON Canada; ^6^ Harquail Centre for Neuromodulation, Sunnybrook Research Institute, Toronto, ON Canada

## Abstract

**Background:**

Personality traits may influence dementia risk by shaping health behaviors, social relationships, and stress responses over the lifespan. According to Reinforcement Sensitivity Theory, individuals vary in their sensitivity to punishment and reward, which is regulated by two systems: the Behavioral Inhibition System (BIS) and the Behavioral Approach System (BAS). The BIS regulates the detection and avoidance of negative outcomes and is associated with increased susceptibility to anxiety and depressive symptoms. Conversely, the BAS promotes approach toward reward‐related stimuli and is linked to excitement and happiness. The relationship between BIS/BAS and cognitive decline among multi‐ethnic populations remains unexplored. This study examined associations between BIS/BAS activity and subjective cognitive decline (SCD), and whether these relationships differed across Chinese, South Asian, and White adults.

**Method:**

Participants were 139 cognitively unimpaired Chinese (*n* =49), South Asian (*n* =39), and White (*n* =51) adults, aged 55‐85, from the *Canadian Multi‐Ethnic Research on Aging (CAMERA)* study. SCD was measured via the self‐reported Cognitive Functioning Index (CFI). BIS/BAS activity was assessed with four self‐report scales: one BIS subscale and three BAS subscales (Reward Responsiveness, Drive, and Fun Seeking). ANCOVAs evaluated ethnic differences in BIS/BAS scales. Linear regression models examined associations between BIS/BAS scales and SCD. Models were adjusted for age, sex, and years of education.

**Result:**

Compared to White participants, Chinese participants had higher BAS Drive scores (*β*=‐1.20, *p*=0.03). No significant group differences were observed for the remaining BIS/BAS scores (*p*>0.05). Ethnicity moderated the associations between BIS/BAS scores and SCD. Specifically, higher BIS, BAS Drive, and BAS Fun Seeking scores were associated with greater SCD in Chinese and South Asian participants, but not in White participants (Table 1). Conversely, higher BAS Reward Responsiveness was associated with lower SCD in White participants, but not in Chinese and South Asian participants (Table 1).

**Conclusion:**

BIS/BAS activity may influence SCD in distinct ways across ethnic groups. Higher BIS, BAS Drive, and Fun Seeking may indicate greater vulnerability to SCD in collectivist cultures, whereas BAS Reward Responsiveness may be protective in more individualistic cultures. These results underscore the importance of accounting for cultural context when examining the interplay between personality traits and cognitive aging.